# Prevalence of onchocerciasis, attitudes and practices and the treatment coverage after 15 years of mass drug administration with ivermectin in the Tombel Health District, Cameroon

**DOI:** 10.11604/pamj.2020.35.107.16036

**Published:** 2020-04-08

**Authors:** Sharon Mumah Nyagang, Samuel Nambile Cumber, Jerome Fru Cho, Elsie Indah Keka, Claude Ngwayu Nkfusai, Emerson Wepngong, Joyce Mahlako Tsoka-Gwegweni, Eric Bertrand Fokam

**Affiliations:** 1 Department of Microbiology and Parasitology, Faculty of Science, University of Buea, Buea, Cameroon; 2 Cameroon Baptist Convention Health Services (CBCHS), Yaounde, Cameroon; 3 Office of the Dean, Faculty of Health Sciences, University of the Free State, Bloemfontein, South Africa; 4 Centre for Health Systems Research and Development, University of the Free State, Bloemfontein, South Africa; 5 School of Health Systems and Public Health, Faculty of Health Sciences, University of Pretoria, Pretoria, South Africa

**Keywords:** Onchocerciasis, prevalence, ivermectin, treatment coverage, attitudes, practices, Tombel health district, nodules, microfilaria, community directed treatment with ivermectin

## Abstract

**Introduction:**

Onchocerciasis is an infection caused by *Onchocerca volvulus*. It affects 37 million people of which 99% are in Africa. This study assessed the prevalence of onchocerciasis after 15 years of mass drug administration with ivermectin.

**Methods:**

This was a population based cross sectional study. Questionnaires covering participants' identity and attitudes and practices of community respondents towards ivermectin were administered. The treatment coverage was obtained by review of records of mass drug administration from 1999 to 2015. The epidemiological evaluation of infection status was done by parasitological examination of skin snips and nodule palpation in individuals in five health areas of the district.

**Results:**

A total of 400 participants were randomly selected. Of these, 56.0% were males, 62.0% single, 59.5% farmers and 98.0% Christians. Participants with good attitudes towards community directed treatment with ivermectin made up 80.5% while 47.8% of the participants had good practice. The highest treatment coverage achieved was 88.0% in 2010 while lowest was 57.0% in 2002. Less than 2% had microfilaria and 6.0% had nodules. There was no statistically significant difference in the prevalence of microfilaria with respect to age. There was a statistically significant difference in the distribution of nodules (χ^2^=73.6, p=0.001) among the different age groups. The greatest rate of infection (2.1%) was among farmers.

**Conclusion:**

This study showed that the prevalence reduced compared to other prevalence studies in Cameroon. The study area was hypo-endemic for onchocerciasis.

## Introduction

Onchocerciasis is an infection caused by the nematode *Onchocerca volvulus*. Humans acquire onchocerciasis through the bite of an infected female black fly in the genus *Simulium*. Since the fly larvae develop in fast-flowing water, onchocerciasis is mostly common along rivers and is also referred to as river blindness. The disease affects rural communities and is a major cause of blindness and skin disease in endemic areas with serious socioeconomic consequences [1]. It affects about half a million people in sub-Saharan Africa. Onchocerciasis also causes skin disease with acute and chronic dermatitis, lichenification, atrophy, depigmentation and severe itching. It is estimated to affect over 37 million people worldwide. Onchocerciasis has not caused a single death but the global burden is 987,000 disability adjusted life years (DALYs). The disease has been found to significantly reduce the lifespan of infected people [2]. In 1995, the WHO launched the African Programme for Onchocerciasis Control (APOC) that was intended to eliminate onchocerciasis from the African continent through Community Directed Treatment with Ivermectin (CDTI) in the next 12years [3]. However, following the 2006 and 2007 progress report, the APOC was extended to 2015.

The CDTI strategy has been very successful in ensuring sustained high treatment coverage and by the year 2010 some 75 million people at risk were treated annually with ivermectin in the APOC countries [4]. Rapid Epidemiological Mapping for Onchocerciasis (REMO), has shown that the disease is endemic in Cameroon [5]. The issue of serious adverse events or side effects (SAEs) due to ivermectin in areas co-endemic for loiasis has been known for some time. The only current control measure is CDTI. In this perspective, the purpose of this study was to assess the prevalence of onchocerciasis in Tombel Health District (THD) where CDTI has been carried out for 15 consecutive years, in order to evaluate the impact of ivermectin treatment. To assess the impact of mass drug administration with ivermectin over 15 years, the prevalence in this study was compared to the prevalence in other areas of Cameroon such as the Fundong health district [6] and the West region of Cameroon [7].

## Methods

**Study design:** the study was a population based cross sectional study designed to assess the prevalence of onchocerciasis after fifteen years of mass drug administration (MDA) with ivermectin and to determine the attitudes and practices towards ivermectin and the treatment coverage with ivermectin. This involved collection of both qualitative and quantitative data from five randomly selected health areas namely: Ebonji, Nyasoso, Baseng, Ndibenjock and Tombel. Qualitative data collection involved administration of pre-tested structured questionnaires covering participants´ demographic factors, attitudes and practice towards ivermectin and the review of treatment records of mass drug administration with ivermectin from 1999 to 2015 to obtain the treatment coverage. For quantitative data collection, the areas of the participants´ body that are most likely to contain subcutaneous nodules were felt. While this technique did not definitively demonstrate onchocerciasis, it was a good indication that further tests should be run to confirm this diagnosis. Parasitological examination was then carried out in five health areas to detect infection. Two skin biopsies were collected from each participant who consented, from the shoulder and the waist using a sterile surgical blade. The skin snips were placed on microscope slides with normal saline and then examined under light microscope (x10) for any microfilaria. The proportion of participants with microfilaria was then calculated and reported as the prevalence.

**Study period:** the study was conducted from November 2017 to February 2018.

**Setting:** Tombel health district (THD) is divided into seven health areas and has a total population of 63267. Tombel is a town in the Southwest region of Cameroon, located in the rain forest area of Southwest region and northern part of the valley of the Mungo river [8]. The presence of forested areas whether uniform or in patches with slow or fast flowing streams, high temperatures, rainfall and human activities favour the development of *Onchocerca* vectors and are key determinants of onchocerciasis occurrence.

**Study participants:** a total number of 400 participants were randomly selected from the five health areas.

**Sample size determination:** sample size was determined based on the Taro Yamane's approach [9].

$$n=\frac{N}{1+N{\left(e\right)}^{2}}$$

where, n=the expected sample size, N=finite population out of which the sample size is drawn, e=level of precision. The target population for this study was 52575 [10]. n=35683/1+52575(0.05)2; n=396 participants.

### Selection criteria

**Inclusion criteria:** those from 20 years and above who signed the consent form and those below 20 years whose guardian signed the consent form and assent obtained from these children were included in the study. A qualified subject must have lived in the area for at least five years prior to the study.

**Exclusion criteria:** children below 5 years and those who refused to sign the consent form were excluded.

**Statistical analysis:** statistical analysis was carried out to determine the prevalence of onchorceciasis. Data collected was entered in excel and coded properly to allow for proper analysis. Data was checked for completeness, outliers, missing values, using SPSS version 22 according the different variables. Using the Chi square test, data obtained in continuous form was summarized as mean and standard deviation. Data obtained in categorical form was summarized as percentages and proportions. Data was displayed in the form of bar graphs and contingency tables.

**Ethical considerations:** administrative authorizations were obtained from the Regional Delegation of Public Health for the Southwest region, the district medical officer of the Tombel health district and the director of Tombel district hospital. Ethical clearance was sought and obtained from the Institutional Review Board, University of Buea. Issues about the autonomy, protection and confidentiality of the subjects were fully respected.

## Results

**Socio-demographic characteristics of the study participants:** a total of 400 participants aged 5-92 years were recruited for the study. The majority were in the age range 21 to 40 with mean age of 37.65±19.056 years (Table 1). Most of the participants were males and majority were single. Farmers represented a higher percentage. A greater proportion of participants were Christians.

**Table 1:** Socio-demographic characteristics of 400 study participants recruited from the community, THD, 2016

Characteristics	Number
**Age group**	
5-20	83(20.8)
21-40	157(39.3)
41-60	99(24.8)
60+	61(15.3)
**Health area**	
Ebonji	80(20)
Nyasoso	80(20)
Baseng	80(20)
Ndibenjock	80(20)
Tombel	80(20)
**Sex**	
Males	224(56.0)
females	176(44.0)
**Marital status**	
Single	248(62.0)
Married	152(38.0)
**Profession**	
Farmer	238(59.5)
Others	162(40.5)
**Education level**	
No formal education	35(8.8)
Primary	253(63.2)
Secondary and above	112(28.0)
**Religion**	
Christian	392(98.0)
Muslim	8(2.0)

**Attitude and practice of community respondents about CDTI in the THD:** among the respondents, 329 (82.3%) perceived CDTI as very useful programme (Table 2), but 52 (13.0%) perceived the programme as partially useful (Table 2). For responsibility of the programme, 361 (90.8%) knew their responsibility (Table 3) and for sustainability of this programme, 272 (68.0%) recommended continuous drug supply, while 106 (26.5%) recommended incentive for community-directed distributors (CDDs) (Table 3). The majority (79.0%) responded that all eligible family members took the drug annually, whereas 84 (21.0%) responded that family members missed at least one round of the drug due to health problems, pregnancy and absence during drug distribution (Table 3). To facilitate the late memory to the reaction of the drug, the respondents were asked when they did receive the last treatment and 340 (85%) of the respondents responded last year (Table 3).

**Table 2:** Attitude of community respondents about CDTI in the THD

Indicative question on attitude	Response	Number (%)
**How do you perceive CDTI (mectizan)? (A)**	Very useful	329(82.3)
	Partially useful	52(13.0)
	Not useful	9(2.3)
	I don’t know	10(2.5)
**How does your family perceive CDTI? (A)**	Very useful	340(85.0)
	Partially useful	45(11.3)
	Not useful	5(1.3)
	I don’t know	10(2.5)
**Do you think the programme on controlling onchocerciasis (River blindness) is effective? (A)**	Yes	359(89.8)
	No	22(5.5)
	I don’t know	19(4.8)
**Why did he/she or you miss treatment? (A)**	Pregnancy	21(24.7)
	Health problem	35(41.2)
	Absent during distribution	26(30.6)
	No reason	3(3.5)
**Do you know who interrupted the treatment in the village (A)**	Yes	111(27.8)
	No	289(72.2)
**If your answer is yes, what was the reason to interrupt the treatment (A)**	Lack of good case	79(71.2)
	Side effect of the drug	30(27.0)
	Management of side effect.	2(1.8)

**Table 3:** Practice of community respondents about CDTI in the THD

Indicative question on practice	Response	Number (%)
**What is your contribution in the CDTI? (P)**	Taking the drug continuously	361(90.8)
	I don’t know	39(9.2)
**What do you recommend to continue the programme? (P)**	Drug supply	272(68.0)
	Transport	13(3.3)
	Incentive for CDDs	106(26.5)
	No comment	9(2.3)
**Have all eligible family members received ivermectin (mectizan)? (P)**	Yes	316(79.0)
	No	84(21.0)
**If your answer is no, who missed the treatment? (P)**	Wife	35(41.2)
	Husband	22(25.9)
	Children	28(32.9)
**How many times did he/she or you miss the treatment? (P)**	One time	46(54.1)
	Two times	14(16.5)
	More than two times	25(29.4)
**When did you receive your Last Treatment? (P)**	Last year	340(85)
	Treatment before two years	60(15)
**Did the drug have any side effects (P)**	Yes	107(26.8)
	No	285(71.2)
	I don’t know	8(2)

P = Practice

**Figure 1:**
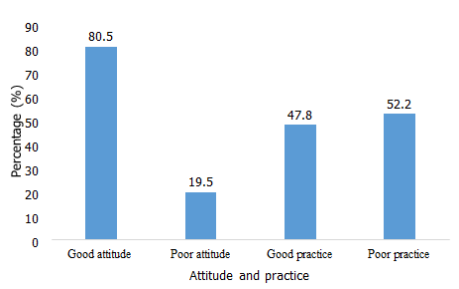
Proportion of participants with microfilaridermia and nodules (blue: percentage)

**Attitude and practice on the CDTI programme:** participants with good attitude and good practice recorded 80.5% and 47.8% (Figure 1) respectively while participants with poor attitude and poor practice recorded 19.5% and 52.2% respectively (Figure 1).

**Figure 2:**
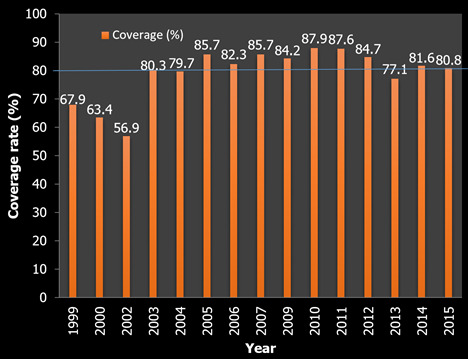
Coverage of mass drug administration with ivermectin in Tombel health district (orange: percentage)

**Coverage of mass drug administration with ivermectin in Tombel health district:** the highest treatment coverage was achieved in 2010 followed by 2011. The lowest treatment coverage was recorded in 2002. Data on treatment coverage for 2001 and 2008 could not be found. The annual treatment coverage recommended by WHO (80%) was not achieved in 1999, 2000, 2002, 2004 and 2013 (Figure 2).

**Proportion of participants with microfilaridermia and nodules:** the overall prevalence of microfilaredermia and nodules was 1.5% and 6.0% respectively.

## Discussion

This study assessed the parasitological status, the treatment coverage and the attitudes and practices of community respondents towards ivermectin in the THD of Cameroon after fifteen years of MDA with ivermectin. The age group of 61+ years had the highest rate of onchocerciasis. This could be because this age group was mostly constituted of farmers. A similar observation to the findings of this study has been reported [11]. Participants with good attitudes towards CDTI made up 80.5% while 47.8% of the participants had good practices towards the CDTI. The highest treatment coverage was 88.0% achieved in 2010 while the lowest treatment coverage was 57.0%, recorded in 2002. Nodule prevalence was 6% while the prevalence of microfilaria was 1.5%. The prevalence in this study was lower than reported in other areas of Cameroon such as the Fundong health district [5] and the West region of Cameroon [6]. Elimination is considered attained when the microfilaria prevalence in skin snips is less than 5% in sampled communities.

This study showed that the study area was hypo-endemic for onchocerciasis, following fifteen years of continuous treatment with ivermectin. The prevalence of onchocerciasis was higher among males than female subjects. The differences in infection rate with regards to sex could be due to endemicity [12], occupational exposure and susceptibility of individual [13]. Males work longer in the farm, and with bare body, than the females thereby making them more prone to the bite of the black flies. The findings in this study differ from another report [14] in Ovia, Northeast of Edo State Nigeria where females had 93% onchocerciasis infection and males 74.5%. The prevalence of nodules was 6%. The results of this survey suggested that change has occurred in the prevalence of onchocerciasis nodules. The prevalence of *Onchocerca* nodules was low and a similar observation has been reported [15]. According to Molyneux [16], 65%-80% coverage is necessary for significant and persistent regression in morbidity.

The treatment coverage in the health areas was at an average of 74.1% with combined range of 73.2%-78.3%. The average treatment coverage as well as the range were within the acceptable rate. This was expected considering the good record keeping observed in the treatment registers. The treatment history did not vary from one health area to another. It was evident that ivermectin treatment was immediately introduced in all health areas at the same time and as a result, the number of treatment rounds as at the same time of the study was the same. This was an indication of consistent annual treatment and availability of sufficient ivermectin. The findings in this study differ from another report [17] in Kaduna State, Nigeria. In this study, the full participation of the community could be largely affected by the drug reaction, lack of good case, management of side effect, absence during drug distribution and health problems. This observation is consistent with the study conducted in Okpuje, an endemic community in Edo State, Nigeria [18]. In this study, almost all the participants knew ivermectin is very important for significant reduction in morbidity and took the drug properly.

This observation is consistent with studies conducted in a hyper-endemic community in Imo State [19] and Edo State [20] in Nigeria and in Ethiopia [21]. However, a few individuals interrupted the treatment due to fear of drug related adverse reactions which is similar to the findings of studies conducted in Sequa area, Southwest Ethiopia [21] and the hyper-endemic community of Edo State, Nigeria [20]. Generally, majority of the study participants had good attitudes about the CDTI programme (80.5%). This finding differs from the findings of the study conducted in the Quara district Northwest Ethiopia [22]. Greater percentage of the participants had poor practices compared to good practices. This finding is consistent with the findings of the study conducted in the Quara district, Northwest Ethiopia. The poor practices were probably due to the lack of health education at the community level and the CDDs might not have been properly trained on the CDTI programme due to negligence of health extension workers to supervise the CDDs in delivering health education.

## Conclusion

This study showed that, the prevalence of nodules and microfilaridermia reduced from >40% and >5% to 6.0% and 1.5% respectively following 15 years of continuous treatment with ivermectin. The treatment coverage in the health areas was at an average of 74.1% with combined range of 73.2%-78.3%.

### What is known about this topic

- Impact of the CDTI programme towards eradication of the onchocerciasis;
- The efficacy of the drug as well as the attitude and practice of community participants towards the drug;
- Prevalence studies on onchocerciasis in areas like the West and Southwest regions of Cameroon.

### What this study adds

- Shows a high treatment coverage (74.1%) with ivermectin in the Tombel Health District.
- Shows high rate of poor practice (52.2%) towards ivermectin despite the endemicity of onchorcerciasis;
- Demonstrates a need for training of CDDs on the CDTI programme and health education at the community level.

## Competing interests

The authors declare no competing interests.
